# Cardiovascular risk of sitagliptin in treating patients with type 2 diabetes mellitus

**DOI:** 10.1042/BSR20190980

**Published:** 2019-07-16

**Authors:** De-kang Zeng, Qian Xiao, Fa-qi Li, Yu-zhi Tang, Chao-li Jia, Xue-wen Tang

**Affiliations:** 1 Department of Geriatrics, People’s Hospital of Chongqing Banan District, Chongqing 401320, China; 2 Department of Geriatrics, The First Affiliated Hospital of Chongqing Medical University, Chongqing, China; 3 Institute of Reproductive and Genetic, Chongqing Health Center for Women and Children, Chongqing 400010, China; 4 Department of Cardiology, People’s Hospital of Chongqing Banan District, Chongqing 401320, China

**Keywords:** cardiovascular safety, sitagliptin, type 2diabetes mellitus

## Abstract

Patients with type 2 diabetes mellitus (T2DM) have a very high risk of cardiovascular related events, and reducing complications is an important evaluation criterion of efficacy and safety of hypoglycemic drugs. Previous studies have shown that the dipeptidyl peptidase-4 (DPP-4) inhibitors (DPP4i), such as sitagliptin, might reduce the incidence of major cardiovascular events (MACEs). However, the safety and efficacy of sitagliptin remains controversial, especially the safety for cardiovascular related events. Here, a systematic review was conducted to assess the cardiovascular safety of sitagliptin in T2DM patients. The literature research dating up to October 2018 was performed in the electronic database. The clinical trials about sitagliptin for T2DM patients were included. Two reviewers independently screened literature according to the inclusion and exclusion criteria. The primary outcome was the MACE, and the secondary outcome was all-cause mortality. Finally, 32 clinical trials composed of 16082 T2DM patients were included in this meta-analysis. The results showed that: there was no significant difference between sitagliptin group and the control group on MACE (odds ratio (OR) = 0.85, 95% confidence intervals (CIs) = 0.63–1.15), myocardial infarction (MI) (OR = 0.66, 95% CI = 0.38–1.16), stroke (OR = 0.83, 95% CI = 0.44–1.54) and mortality (OR = 0.52, 95% CI = 0.26–1.07). These results demonstrated that sitagliptin did not increase the risk of cardiovascular events in patients with T2DM.

## Introduction

Type 2 diabetes mellitus (T2DM) is a progressive disease characterized by insulin deficiency and insulin resistance. It is associated with a high risk of microvascular, cardiovascular and other complications (such as depression, a mental disorder with unclear pathogenesis) [1–4]. Most interventions for T2DM are designed to control the blood glucose level. However, these interventions pay little attention to other risk factors and rarely meet the multifaceted needs of patients with T2DM [5,6]. Currently, the main challenges of T2DM treatment include: maintaining tight glycemic control, minimizing the risk of hypoglycemia, controlling cardiovascular risk factors (such as blood pressure and serum lipid concentrations), and reducing or controlling weight. Diabetic patients suffer from a high rate of cardiovascular events. Therefore, the European Society of Cardiology (ESC) Guidelines on diabetes, pre-diabetes and cardiovascular disease (CVD) emphasize the need for a stringent approach in treating patients with diabetes, suggesting the importance of reducing the cardiovascular events [7,8].

Dipeptidyl peptidase‐4 (DPP‐4) inhibitors (DPP4i) are a pharmacological class of oral hypoglycemic drugs, which could prolong the action of the incretin peptide hormones glucagon-like peptide (GLP)-1 and glucose-dependent insulinotropic polypeptide (GIP) by inhibiting their breakdown. Previous study suggested that DPP‐4 inhibition might possibly have a beneficial cardiovascular effect in humans [9]. GLP-1 could suppress the release of glucagon from the pancreas. In addition, GLP-1 and GIP could also preserve or enhance β-cell function [10,11]. The levels of intact GLP-1 and GIP could decrease rapidly, due to enzymatic inactivation (mainly DPP-4) and renal clearance [12]. Sitagliptin, a potent and selective DPP4i, is specifically designed to extend the inhibition of DPP-4 enzyme. It could improve glycemic control through enhancing the action of GLP-1and GIP [13]. Fonseca et al. [14] reported that sitagliptin could improve glycemic control and be generally well tolerated by patients with T2DM.

Sitagliptin could better reduce the levels of low-density lipoprotein cholesterol and total cholesterol compared with pioglitazone [15]. Cornel et al. [16] reported that sitagliptin had no clinically significant impact on cardiovascular outcomes. However, whether sitagliptin has clinically significant impact on decreasing cardiovascular events or not is still controversial. Therefore, we conducted this work to assess the cardiovascular risk of sitagliptin in treating patients with T2DM.

## Materials and methods

### Study selection

The literature research was conducted using the following scientific and medical databases: international databases (PubMed, Embase, Web of Science and Medline) and Chinese databases (CBM-disc, CNKI), which was up to October 2018. The search terms used were ‘sitagliptin’, ‘diabetes’, ‘T2DM’ and ‘type 2 diabetes mellitus’. To avoid omitting relevant articles, no language was imposed, and reference documents listed in the included articles were also researched.

Among the articles identified in the initial research, only those meeting the following criteria were selected for subsequent analysis: (i) patients with T2DM; (ii) compared sitagliptin with placebo or active drugs (oral hypoglycaemic agents and/or insulin) different from other DPP4i; (iii) clinical trials with duration of at least 24 weeks. Potential articles meeting any one of the following criteria were excluded: (i) clinical trials with duration shorter than 24 weeks; (ii) patients with nondiabetic or type 1 diabetic; (iii) reviews, case reports and duplicate reports.

### Data extraction and quality assessment

Two authors (De-kang Zeng and Yu-zhi Tang) of the present study served as reviewers to independently verify all potentially suitable clinical trials based on the aforementioned criteria, and extracted data subsequently. Any disagreement was resolved by the third reviewer. For all included articles, results reported in papers were used as the primary source of information. The primary outcome was major cardiovascular events (MACEs), including cardiovascular death, nonfatal myocardial infarction (MI) and stroke, and the secondary outcome was all-cause mortality.

Two authors (De-kang Zeng and Yu-zhi Tang) of the present study served as reviewers to independently assess the quality of each eligible study according to the Cochrane Collaboration criteria [17]. Bias risk was determined by: (i) randomization quality, (ii) allocation concealment, (iii) blinding of outcome assessment, (iv) incomplete reporting of outcome data, (v) similarity in baseline clinical characteristics. Studies with three or more bias risks were excluded from the meta-analysis.

### Statistical analysis

This meta-analysis was conducted according to the recommendations of Sacks et al. [18]. Dichotomous data were preferred for clinical reasons. Baseline scores, standard deviations (SDs), and end point means were used to estimate the number of responsive patients under the condition that dichotomous efficacy outcomes were absent [19]. To perform a clinically sound analysis, a worst-case scenario analysis of drop-outs was used, under the assumption that all such patients did not respond to treatment [20]. The weighting of each study was performed according to the number of samples in each study. The meta-regression was used here to examine the impact of moderator variables, such as sex ratio and age, on the study effect size. Statistical analyses were performed using RevMan 5.3 (Cochrane Information Management System) and STATA software 8.0 (Stata Corporation, College Station, TX, U.S.A.). Heterogeneity was assessed using the Q statistic and *I^2^* [21]. A *P*-value <0.1 or *I^2^* value >50% indicated that there was significant statistical heterogeneity among the included studies. If there was heterogeneity, the pooled odds ratio (OR) and 95% confidence intervals (CIs) were calculated by Mantel–Haenszel fixed-effects model; otherwise, the random-effects model was used [22]. Finally, funnel plots and Egger’s test were used to assess the potential presence of publication bias. This research was performed independently of any funding, as part of the institutional of the investigators.

## Results

### Selected studies

**Figure 1:**
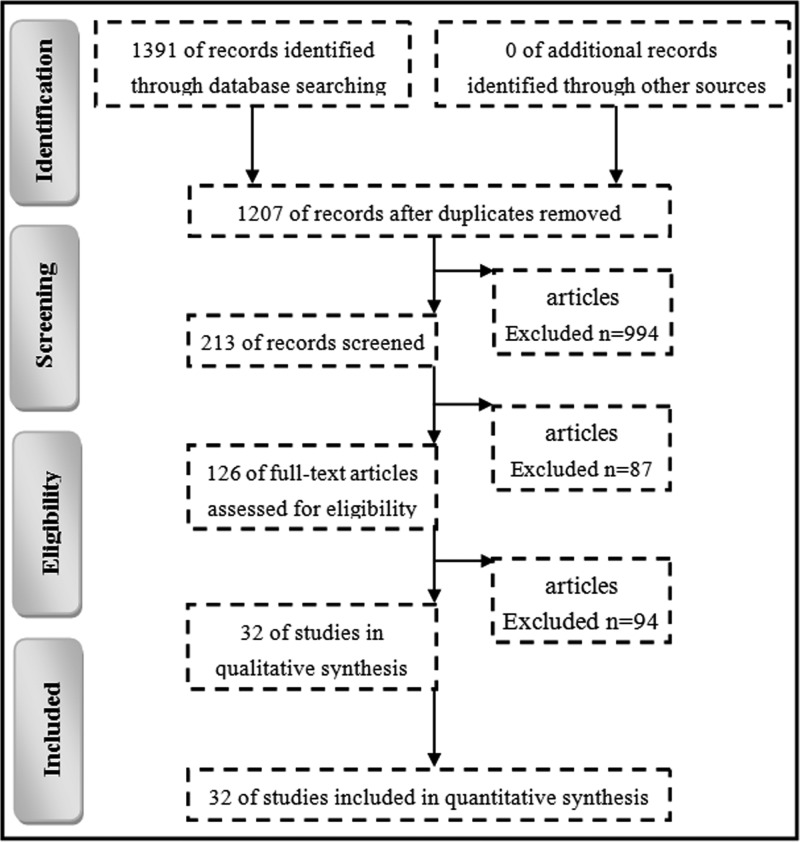
Flow chart of the present study

The literature search yielded 1391 potentially relevant studies. However, only 32 studies met all the inclusion and exclusion criteria mentioned above [14,15,23–52], and could be used for subsequent analysis (Figure 1). Among these studies, 12 studies were active comparator controlled and 20 studies were placebo controlled. Four studies did not report any adverse events and were excluded from the subsequent analysis. A total of 16082 patients with T2DM were included. Of these, 8536 patients were treated with sitagliptin and the remaining 7546 patients were treated with placebo or active drugs different from other DPP4i. Table 1 presented the detailed characteristics of the included studies.

**Table 1:** Baseline characteristics of trials included in the meta-analysis

First author (year)	Sitagliptin					Control					Duration (weeks)
	*n*	Male	Female	Age (years)	BMI	*n*	Male	Female	Age (years)	BMI	
Henry (2014)	922	524	398	52.1 ± 8.7	NR	693	388	305	51.5 ± 9.2	NR	54
Chan (2008)	65	31	34	68.9 ± 9.8	26.5 ± 4.0	26	16	10	65.3 ± 9.7	26.9 ± 4.9	54
Ferreia (2013)	211	125	86	64.8 ± 10.6	26.5 ± 4.8	212	116	96	64.3 ± 9.2	27.0 ± 4.8	54
Williams (2010)	551	265	286	54.1 ± 9.1	31.2 ± 6.5	540	259	281	54.7 ± 9.9	32.0 ± 6.4	104
Charbonnel (2006)	464	259	205	54.4 ± 10.4	30.9 ± 5.3	237	141	96	54.7 ± 9.7	31.5 ± 4.9	24
Raz (2006)	411	214	197	55.4 ± 9.2	31.9 ± 5.3	110	69	41	55.5 ± 10.1	32.5 ± 5.2	44
Aschner (2006)	488	253	235	54.1 ± 9.8	30.3 ± 5.3	253	130	123	54.3 ± 10.1	30.8 ± 5.5	24
Reasner (2011)	625	353	272	49.4 ± 10.5	32.9 ± 7.2	621	356	265	50.0 ± 10.5	33.7 ± 7.8	44
Visboll (2010)	322	157	165	58.3 ± 9.1	31 ± 5	319	169	150	57.2 ± 9.3	31 ± 5	24
Hermansen (2007)	222	117	105	55.6 ± 9.6	31.2 ± 6.3	219	117	102	56.5 ± 9.6	30.7 ± 6.3	24
Monteverde (2011)	244	152	92	50.5 ± 10.9	30.3 ± 5.2	248	148	100	51.7 ± 10.1	29.4 ± 5.2	40
Dobs (2013)	170	96	74	54.4 ± 8.8	30.1 ± 6.2	92	55	37	54.8 ± 9.5	30.8 ± 5.6	54
Pratley (2012)	219	120	99	55.0 ± 9.0	32.6 ± 5.4	446	228	218	55.4 ± 9.4	32.8 ± 5.1	78
Arechavaleta (2011)	516	284	232	56.3 ± 9.7	29.7 ± 4.5	519	279	240	56.2 ± 10.1	30.2 ± 4.4	30
Yang (2012)	197	92	105	54.1 ± 9.0	25.3 ± 3.1	198	108	90	55.1 ± 9.8	25.3 ± 3.6	24
Bergenstal (2010)	166	86	80	52 ± 11	32 ± 5	325	168	157	52.5 ± 10	32 ± 5.5	26
Aschner (2010)	528	217	311	56.3 ± 10.7	30.7 ± 4.7	522	194	328	55.7 ± 10.3	30.9 ± 4.9	24
Wainstein (2012)	261	143	118	52.4 ± 10.7	30.3 ± 6.1	256	134	122	52.2 ± 11.0	29.6 ± 5.5	32
Seck (2010)	588	336	252	57.6 ± 8.5	30.9 ± 4.8	584	367	217	57.0 ± 9.1	31.3 ± 5.0	104
Yoon (2011)	261	137	124	50.2 ± 10.2	29.7 ± 5.1	259	145	114	51.7 ± 11.2	29.6 ± 5.2	24
Fonseca (2013)	157	97	60	55.7 ± 8.7	29.9 ± 5.2	156	98	58	56.4 ± 9.4	30.0 ± 5.2	26
Barzilai (2011)	102	48	54	71.6 ± 6.1	30.8 ± 5.9	104	49	55	72.1 ± 6.0	31.1 ± 7.2	24
Yoon (2012)	164	86	78	51.4 ± 10.0	29.7 ± 4.8	153	90	63	52.3 ± 11.5	29.9 ± 5.3	24
Raz (2008)	96	49	47	53.6 ± 9.5	30.4 ± 5.3	94	39	55	56.1 ± 9.5	30.1 ± 4.4	30
Rosenstock (2006)	175	93	82	55.6 ± 10.4	32.0 ± 5.2	178	103	75	56.9 ± 11.1	31.0 ± 5.0	24
Stein (2014)	10	6	4	61.3 ± 8.2	34.3 ± 3.3	11	8	3	60.8 ± 7.6	32.7 ± 2.7	48
Zang (2016)	184	117	67	51.4 ± 11.0	27.2 ± 4.0	183	102	81	51.7 ± 10.7	27.3 ± 3.4	26
Kim (2017)	147	81	66	54.8 ± 8.5	25.2 ± 2.7	145	84	61	53.1 ± 9.2	25.0 ± 2.8	30
Ji (2016)	120	74	46	51.7 ± 10.2	26.0 ± 3.5	126	69	57	52.6 ± 9.5	26.0 ± 3.7	24
Ba (2016)	249	117	132	57.5 ± 9.5	25.4 ± 3.2	249	132	117	56.5 ± 9.3	25.3 ± 3.2	24
Shankar (2016)	234	130	104	58.6 ± 8.4	25.9 ± 3.0	233	116	117	56.7 ± 9.1	26.1 ± 2.9	24
Duan (2016)	105	59	46	50.32 ± 3.21	27.10 ± 1.73	103	55	48	48.88 ± 2.91	27.32 ± 1.81	24

Abbreviations: BMI, body mass index; *n*, number; NR, not available.

### MACEs

**Figure 2:**
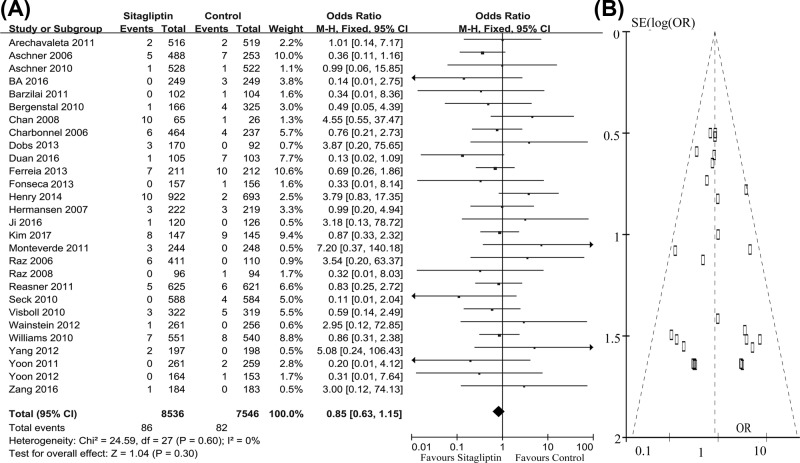
Number of patients with MACEs in two groups (**A**) Meta-analysis showed the non-significant difference on this outcome; (**B**) funnel plots showed no potential publication bias.

MACEs were reported in 28 studies. In these clinical trials, 86 of 8536 patients receiving sitagliptin and 82 of 7546 comparator patients reported MACE, respectively. The pooled OR was 0.85 (95% CI = 0.63–1.15, z = 1.04, *P* 0.30), indicating that there was no statistical difference between those two groups on the incidence of MACE. The risk of MACE was not significantly increased in patients receiving sitafliptin (Figure 2A). The funnel plot of these studies appeared to be closely symmetrical, which indicated that there was no publication bias (Figure 2B). Meanwhile, the results of Egger’s test (*P*=0.54) also showed that the outcome was not influenced by the potential publication bias. Meanwhile, the results of meta-regression showed that our effect size was not influenced by these moderator variables.

The study period of two included studies was 104 weeks. Therefore, the sensitivity analysis was conducted by excluding these two studies. The new pooled OR was 0.89 (95% CI = 0.65–1.23, z = 0.69, *P*=0.49), which was similar to the original results. Meanwhile, the subgroup analysis was conducted according to the study period (≥54 and <54 weeks). The pooled OR of these studies with study period ≥54 weeks was 1.16 (95% CI = 0.69–1.96, z = 0.57, *P*=0.57), and the pooled OR of these studies with study period <54 weeks was 0.73 (95% CI = 0.50–1.05, z = 1.69, *P*=0.09). These results indicated that our conclusion was not significantly influenced by the different treatment periods. The subgroup analysis was also conducted according to the different sorts of control groups (sitagliptin vs. placebo and sitagliptin vs. active drugs). The pooled OR of sitagliptin vs. placebo was 0.73 (95% CI = 0.49–1.09, z = 1.55, *P*=0.12), and the pooled OR of sitagliptin vs. active drugs was 1.05 (95% CI = 0.66–1.68, z = 0.22, *P*=0.83). These results indicated that our conclusion was not significantly influenced by the different sorts of control groups.

### MI

**Figure 3:**
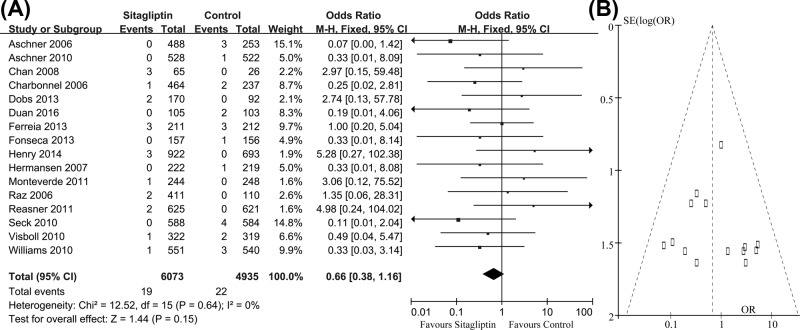
Number of patients with MI in two groups (**A**) Meta-analysis showed the non-significant difference on this outcome; (**B**) funnel plots showed no potential publication bias.

MI was reported in 16 studies. In these clinical trials, 19 of 6073 patients receiving sitagliptin and 22 of 4935 comparator patients reported MI, respectively. The pooled OR was 0.66 (95% CI = 0.38–1.16, z = 1.44, *P*=0.15), indicating that there was no statistical difference between those two groups on the incidence of MI. The risk of MI was not significantly increased in patients receiving sitafliptin (Figure 3A). The funnel plot of these studies appeared to be closely symmetrical, which indicated that there was no publication bias (Figure 3B). Meanwhile, the results of Egger’s test (*P*=0.47) also showed that the outcome was not influenced by the potential publication bias.

### Stroke

**Figure 4:**
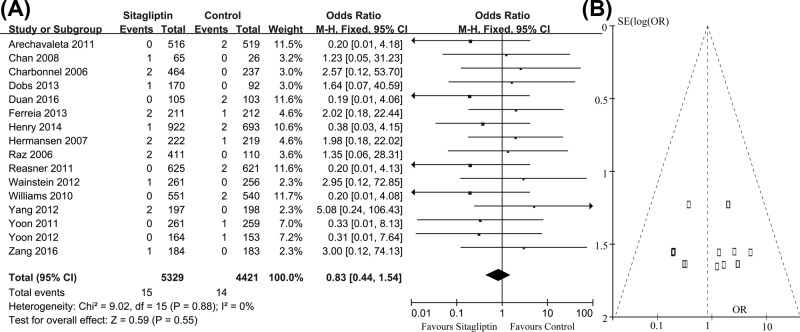
Number of patients with stroke in two groups (**A**) Meta-analysis showed the non-significant difference on this outcome; (**B**) funnel plots showed no potential publication bias.

Stroke was reported in 16 studies. In these clinical trials, 15 of 5329 patients receiving sitagliptin and 14 of 4421 comparator patients reported stroke, respectively. The pooled OR was 0.83 (95% CI = 0.44–1.54, z = 0.59, *P*=0.55), indicating that there was no statistical difference between those two groups on the incidence of stroke. The risk of stroke was not significantly increased in patients receiving sitafliptin (Figure 4A). The funnel plot of these studies appeared to be closely symmetrical, which indicated that there was no publication bias (Figure 4B). Meanwhile, the results of Egger’s test (*P*=0.26) also showed that the outcome was not influenced by the potential publication bias.

### Mortality

**Figure 5:**
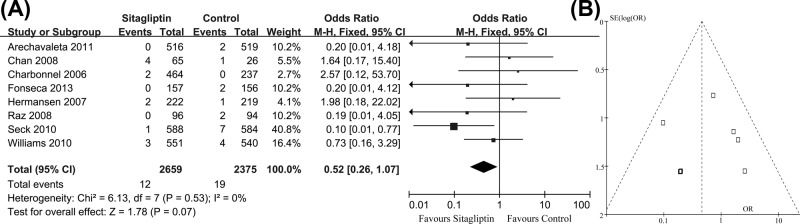
Number of mortality in two groups (**A**) Meta-analysis showed the non-significant difference on this outcome; (**B**) funnel plots showed no potential publication bias.

There were eight studies reported information on mortality (either cardiovascular death or others). In these clinical trials, 12 of 2659 patients receiving sitagliptin and 19 of 2375 comparator patients experienced death, respectively. The pooled OR was 0.52 (95% CI = 0.26–1.07, z = 1.78, *P*=0.07), indicating that there was no statistical difference between those two groups on the incidence of mortality. The risk of mortality was not significantly increased in patients receiving sitafliptin (Figure 5A). The funnel plot of these studies appeared to be closely symmetrical, which indicated that there was no publication bias (Figure 5B). Meanwhile, the results of Egger’s test (*P*=0.30) also showed that the outcome was not influenced by the potential publication bias.

## Discussion

With more than 300 million patients with T2DM in the whole world, as drugs for T2DM, it is not only important to manage the blood glucose but also control the risk of complications, especially decreasing cardiovascular risk [53,54]. So the analysis of serious adverse events in clinical trials is essential for assessing the safety profile of newer drugs. Sitagliptin, as a representative drug of DPP4i, is usually used for treating T2DM. By slowing incretin degradation, DPP4i enhance meal-stimulated active GLP-1 and GIP levels by two- to threefold. When the blood glucose concentration is elevated, GLP-1 and GIP can increase the pancreatic β-cell synthesis and release insulin by increasing intracellular signaling pathways. Sitagliptin could be able to increase the concentration of GLP-1 and GIP [13].

Previous study suggested that sitagliptin could play a role in modifying the high risk of fatal arrhythmias that are inherent in T2DM [55]. However, the efficacy of sitagliptin for T2DM complications remains uncertain. Previous pooled analyses of individual compounds have yielded discordant results, with incidence of cardiovascular events either reduced or unchanged [56,57]. Our results found that the use of sitagliptin would not increase the risk of MACE compared with placebo or other hypoglycemic drugs. The strength of this conclusion was the large number of trials with a large number of patients, and the present meta-analysis confirmed the trend reported in the previous study [57]. We also found that the incidences of MI and stroke were not significantly increased in patients receiving sitagliptin.

Sitagliptin is mostly excreted as unchanged drug by kidney; then, the renal insufficiency would increase the circulating levels of sitagliptin [58]. In contrast, vildagliptin is primarily metabolized by hydrolysis to an inactive compound; then, only approximately 20% of vildagliptin is excreted unchanged [59]. In addition, saxagliptin is mainly metabolized by liver to an active compound; then itself and its metabolites are renally excreted [60]. Many studies have shown that sitagliptin and vildagliptin were comparable on safety and tolerability, both in short-term and long-term treatments [61,62]. Although experience with saxagliptin is more limited, some studies also reported that this drug was tolerable and safe [60,63].

Sodium-dependent glucose transporter 2 (SGLT2) inhibitors are an important emerging class for treating diabetes. Nowadays, six kinds of SGLT2 (tofogliflozin, canagliflozin, empagliflozin, dapagliflozin, ipragliflozin, and luseogliflozin) for diabetes are approved. The common adverse events of SGLT2 include: reproductive system infection, urinary system infection, and ketoacidosis [64]. The common adverse event of sitagliptin is gastrointestinal reaction (such as abdominal pain, diarrhea, nausea, vomiting). As the second- and third-line medication for T2DM, SGLT2 can be used in combination with metformin or other hypoglycemic agents.

The main limitation of the present study was that the dosage of sitagliptin in each study was not the same, which might influence the results of this meta-analysis. However, this limitation was also the general problem for meta-studies to solve. Meanwhile, the main purpose of the existing studies about sitagliptin was to assess the efficacy, tolerability, and safety of blood glucose control. The final conclusion about the influence of sitagliptin on cardiovascular risk in T2DM patients still needs future studies to further investigate, especially the large-scale clinical trials. Finally, a double-blind, controlled, randomized, multi-center study would be needed to highlight the usefulness of sitagliptin and other gliptins. In conclusion, available data from these short- and medium-term trials showed that treatment with sitagliptin did not increase the risk of cardiovascular events in patients with T2DM.

## Abbreviations

CI: confidence interval
DPP-4: dipeptidyl peptidase-4
DPP4i: DPP-4 inhibitor
GIP: glucose-dependent insulinotropic polypeptide
GLP: glucagon-like peptide
MACE: major cardiovascular event
MI: myocardial infarction
OR: odds ratio
SGLT2: sodium-dependent glucose transporter 2
T2DM: type 2 diabetes mellitus
